# Fecal Microbiota Transplantation (FMT) as a Prophylaxis of Necrotizing Enterocolitis (NEC)—Protocol for a Safety Study

**DOI:** 10.3390/ph19030437

**Published:** 2026-03-09

**Authors:** Ewa A. Bieganska, Marek Wolski, Magdalena Zarlenga, Jaroslaw Bilinski, Przemyslaw Kosinski

**Affiliations:** 1 Department of Pediatric Surgery, Medical University of Warsaw, 02-091 Warsaw, Poland; marek.wolski@wum.edu.pl; 2 Department of Neonatology and Rare Diseases, Medical University of Warsaw, 02-091 Warsaw, Poland; magda.cmkp@gmail.com; 3 Department of Hematology, Transplantation and Internal Medicine, Medical University of Warsaw, 02-091 Warsaw, Poland; jaroslaw.bilinski@human-biome.com; 4 Human Biome S.A. Gdańsk, 02-234 Warsaw, Poland; 5 Department of Obstetrics, Perinatology and Gynecology, Medical University of Warsaw, 02-091 Warsaw, Poland; przemyslaw.kosinski@wum.edu.pl

**Keywords:** fecal microbiota transplantation, necrotizing enterocolitis, preterm infants, gut microbiota, neonatal intensive care

## Abstract

**Background/Objectives**: Necrotizing enterocolitis (NEC) is an inflammatory disease with an incidence of about one in 1000 live births, much higher in premature and low birth weight newborns. Intestinal dysbiosis is an important element in the pathogenesis of NEC, and for this reason, experimental models have been used to administer fecal microbiota transplants (FMTs) for prophylaxis and treatment of NEC with very satisfactory results. The primary endpoint of the study is safety, defined as the incidence of adverse events (AEs) and serious adverse events (SAEs) occurring from the time of intervention until hospital discharge, classified according to severity and assessed for relatedness to the intervention. **Methods**: This prospective, single-arm, open-label clinical study will include 20 infants born between 24 0/7 and 36 6/7 weeks of gestation. FMTs will be administered twice as a deep rectal infusion via a Foley catheter. The donors of the material from which the FMT will be prepared will be women in the third trimester of pregnancy. The safety of the therapy will be assessed by comparison with a control group, i.e., 20 patients who will meet the same inclusion criteria and will not meet any of the exclusion criteria, subject to the same hospital observation but without undergoing any medical/therapeutic intervention other than the collection of biological material. **Discussion**: The study will provide data on the safety and initial efficacy of FMT in this group of patients, which will allow for further research into the use of this method in the prevention of infections and NEC. Ethics: The study protocol was approved by the Bioethics Committee of the Medical University of Warsaw, Warsaw, Poland (KB/52/2025). All procedures will follow the principles of the Declaration of Helsinki. The results of the study will be submitted for knowledge translation in peer-reviewed journals and presented at national and international pediatric society conferences. Clinical Trial Registration: The study is registered at ClinicalTrials.gov: ID: NCT06333405.

## 1. Introduction

Necrotizing enterocolitis (NEC) is an inflammatory disease affecting one in 1000 live-born newborns. Due to NEC, 2–5% of newborns are hospitalized annually in neonatal intensive care and pathology units. Overall, 85% of all cases of NEC occur in neonates born <32 weeks of gestation (WG) and/or weighing <1500 g. The risk increases with decreasing gestational age. At 22–28 WG, it is 11%; at 28–32 WG, it is 9% and increases to 13% with coexisting congenital heart disease [1,2]. The mortality rate in the general population of patients with NEC is between 15 and 30%, depending on the study, and has remained unchanged for many years [3,4]. The current treatment regimen for NEC is based on symptomatic interventions—cessation of enteral feeding, antibiotic therapy, and, in complicated cases, surgical intervention [1].

According to the most recent studies, intestinal dysbiosis, i.e., altered patterns of intestinal colonization, resulting in abnormal intestinal microbiome composition and microbiota function, plays a very important role in the pathogenesis of NEC and other infectious complications, like neonatal sepsis [5,6,7]. Dysbiosis initiates an unbalanced pro-inflammatory response of the immune system in premature infants, which ultimately leads to a breakdown of the intestinal mucosal barrier, intestinal bacterial translocation, and intestinal necrosis [6,8]. In the study by el Manouni el Hassani et al., it was shown that in both Gram-negative and Gram-positive late-onset sepsis LOS combined, the causative pathogen could be identified in at least one fecal sample collected 3 days prior to LOS onset in the majority of the fecal samples, whereas these pathogens were present in about one-third of control samples [9].

Antibiotic therapy in the mother and newborn, method of delivery, birth weight and birth age have been proven to affect the microbiome [6,10]. Vaginal delivery and breastfeeding ensure the formation of the correct microbiota and microbiome pattern of the gastrointestinal tract in the newborn. Breastfeeding is one of the main preventive factors for NEC, but it is often challenging to achieve for premature newborns due to both neonatal and maternal factors. On the other hand, formula feeding predisposes to the development of NEC through significant changes in the intestinal microbiota [11].

At the same time, meta-analyses provide no strong evidence confirming the preventive effect of standardized probiotics against NEC [12]. Studies indicate a potential positive role of probiotics containing Bifidobacterium and Lactobacillus strains, but appear insufficient to restore the natural microbiota, and, in particular, the microbiome, in the face of multifactorial dysbiosis [13,14].

Despite several meta-analyses on the efficacy of probiotics in reducing mortality and morbidity associated with NEC in premature infants, ESPGHAN (European Society for Paediatric Gastroenterology, Hepatology and Nutrition) and AAP (American Academy of Pediatrics) recommendations for administering probiotics to premature infants are cautious. Despite numerous randomized clinical trials, the optimal strain, dose or combination of bacterial strains that could be routinely recommended for supplementation has still not been clearly identified [15,16,17,18].

Since the important role of intestinal dysbiosis in the pathophysiology of NEC has been discovered, it has been hypothesized that the fecal microbiota transplantation (FMT) procedure, which was proven effective in restoring intestinal eubiosis, may have both prophylactic and therapeutic effects in NEC [19,20,21,22]. Moreover, recent data show that FMT can also decolonize antibiotic-resistant bacteria from the gastrointestinal tract of patients and has immunomodulatory or even anti-infective potential [23,24,25].

Recently published reviews of NEC treatment options reiterate FMT as a potentially very promising method for the prevention and treatment of NEC but emphasize the necessity of validating these findings in a clinical trial setting [26,27].

To the best of our knowledge, FMT has not yet been evaluated in a cohort of premature newborns in this specific prophylactic context. For this reason, we have decided to initiate a clinical trial at our center to test the safety and, potentially, the future effectiveness of this method for preventing NEC.

## 2. Experimental Design

### 2.1. Research Hypothesis

We hypothesize that prophylactic fecal microbiota transplantation in preterm infants is safe and well-tolerated, with an adverse event profile comparable to standard care.

### 2.2. Trial Design

This will be a safety study of FMT for the prevention of NEC, in the form of a prospective, open-label study with continuous recruitment of up to 40 patients in total (20 in the study group, 20 in the control group). Included patients must meet the exact inclusion criteria described below and must not meet any exclusion criteria. The study group will include 10 premature infants born between 28 0/7 and 36 6/7 weeks of gestation, followed by another 10 premature infants born between 24 0/7 and 27 6/7 weeks of gestation, and 20 premature infants in the control group. After completion of this stage (inclusion and treatment of 20 patients + 20 in the control group), the safety of the therapy will be assessed with a description and presentation of all adverse events, their classification and relationship to the intervention.

The safety of the therapy will be assessed by comparison with a control group, i.e., 20 patients who will meet the same inclusion criteria and will not meet any of the exclusion criteria, subject to the same hospital observation but without undergoing any medical/therapeutic intervention other than the collection of biological material (including stool, blood and urine samples for exploratory testing of the gut microbiota).

Recruitment will occur continuously between January 2026 and January 2027. The study is registered at ClinicalTrials.gov (NCT06333405).

### 2.3. Participants, Enrollment, Randomization, and Statistical Methods

The studied population will be the group of premature newborns (born between 24 0/7 and 36 6/7) who are at increased risk of developing NEC and who may benefit most from FMT.

#### 2.3.1. Sample Size

A formal sample size calculation based on power analysis was not performed, as this is a pilot safety study intended to generate preliminary data (estimates of adverse event rates) for a future randomized controlled trial (RCT). A sample size of 20 subjects in the intervention group was determined based on feasibility constraints and standard recommendations for pilot safety studies, following biostatistical consultation [28]. In the first phase, 10 patients born between 28 0/7 and 36 6/7 will be included, followed by 10 patients born between 24 0/7 and 27 6/7 in the second phase.

The control group will include 20 matched controls—patients who meet the same inclusion and exclusion criteria as the study patients.

#### 2.3.2. Allocation

During the safety study phase, we plan to enroll the first 20 patients who meet the inclusion criteria and do not meet the exclusion criteria, who will be admitted to the clinic during the study.

The list will be available to all department physicians who will be involved in the study and trained in study procedures. These will also be the individuals who will decide on the administration of FMT to patients and observe them after the procedure.

Patients will be included in the control and study groups in a 1:1 ratio according to the order of admission to the ward. This means that each subsequent patient included in the study will be included in the opposite group to the previous patient.

The study will not be blinded.

#### 2.3.3. Statistical Analysis

All analyses will be conducted on an intention-to-treat basis.

The primary endpoint is safety, defined as the incidence of adverse events (AEs) and serious adverse events (SAEs) during the observation period.

The proportion of patients experiencing at least one AE or SAE will be compared between groups using Fisher’s exact test. The chi-square test will be applied when its assumptions are met.

The incidence of specific AEs will also be compared using Fisher’s exact test. Relative risks (RRs) with two-sided 95% confidence intervals (CIs) will be calculated.

If the number of events per patient is analyzed, between-group comparisons will be performed using Poisson regression or negative binomial regression, depending on data dispersion.

Time to first AE, if applicable, will be analyzed using Kaplan–Meier estimates and compared using the log-rank test.

Continuous variables will be assessed for normality using the ShapiroWilk test. Normally distributed variables will be compared using Student’s *t*-test, and non-normally distributed variables using the Mann–Whitney U test.

Two-sided tests will be used throughout, with statistical significance set at α = 0.05. Estimates will be presented with two-sided 95% confidence intervals. Analyses will be performed using validated statistical software (e.g., R, Stata, or SAS); the exact software and version will be documented in the final report.

Given the limited sample size, the study is not powered for formal hypothesis testing of small effect sizes. Therefore, interpretation will focus primarily on effect size estimates and their 95% confidence intervals rather than solely on statistical significance. Clinically meaningful differences will be considered in the context of effect magnitude and precision.

#### 2.3.4. Inclusion Criteria

1. Premature babies born naturally or by caesarean section between 24 0/7 and 36 6/7 weeks of pregnancy.
2. At least 24 h after the end of antibiotic therapy, if it is/was used.
3. Up to 6 days of age OR up to 14 days of age in the case of transfer from another facility or temporary contraindications to the administration of microbiota (defined below).
4. Newborns born in the hospital conducting the experiment and outside of it.
5. Premature infants hospitalized in the intensive care unit and neonatal pathology unit during the study period.
6. Signed informed consent of parents/legal guardians for the child’s participation in the experiment in the study group (with the administration of microbiota)—in the version for newborns born between 24 0/7 and 27 6/7 weeks of pregnancy.
  B. Signed informed consent of parents/legal guardians for the child’s participation in the experiment in the study group (with microbiota administration)—version for newborns born between 28 0/7 and 36 6/7 weeks of gestation.
  C. Signed informed consent of parents/legal guardians for the child’s participation in the experiment in the control group (without administration of microbiota)—in the version for the control group.

#### 2.3.5. Exclusion Criteria

1. Congenital defects of the digestive tract preventing normal intestinal transit (atresia of the esophagus, duodenum, rectum, tracheoesophageal fistula).
2. The presence of genetic diseases diagnosed prenatally or perinatally (trisomy, other genetic syndromes).
3. Active antibiotic therapy and the period up to 24 h after its completion.
4. Perforation of the gastrointestinal tract.
5. Food allergy with anaphylactic shock.
6. Participation in another experiment or clinical trial that may affect the final outcome of the planned intervention.
7. Lack of signed informed consent from parents/legal guardians for the child’s participation in the experiment in any of the three groups.

#### 2.3.6. Temporary Exclusion Criteria

Criteria for temporary postponement of microbiota administration (study group) and postponement of the matching time point for administration of intestinal microbiota doses (control group) if, prior to FMT or on day 6 of life, at least one of the following occurs as identical for microbiota administration and up to day 14 of life:

- Oral feeding intolerance: (assessed by the qualifying physician on the day of the planned FMT) if any of the following are present:
  - Painful abdominal distension and/or visible intestinal loops.
  - Blood in the stool.
  - Increased gastric residuals:
    - Two consecutive episodes exceeding >50% of the volume of the previous portion.
    - Two or more consecutive episodes of retention/bilious or duodenal residuals/emesis—when these episodes are not related to anxiety, delayed stool passage, the possibility of swallowing blood during delivery or from damaged nipples, incorrect positioning of the gastric catheter, bleeding from the nasal cavity.
    - Increased gastric residual volume is not defined as a volume of up to 2 mL if the newborn receives a feeding portion of 1–2 mL.
- Suspected NEC based on Bell’s criteria.
- Antibiotic therapy during planned FMT administration.
- Clinical/laboratory/radiological signs of infection—if at least one of the following clinical signs and/or more than one laboratory and/or radiological sign is present:
  - Clinical symptoms of infection:
    - Hemodynamic instability: hypotension, tachycardia, peripheral circulation disorders (according to age norms), thermoregulation disorders, and fever > 38 °C, hypothermia < 36 °C.
    - Apathy, lethargy, and convulsions.
    - Apnea and deterioration of respiratory efficiency.
  - Laboratory symptoms of infection:
    - Elevated inflammation parameters: leukocyte count< 5 and >30 thousand/μL up to 48 h and <5 and >20 thousand/μL > 48 h; left shift in neutrophil differential I:T > 0.2 for >34 h and >0.16 for <34 h.
    - Platelet count < 50 K, coagulopathy.
    - CRP > 0.05 mg/L (normal range < 0.05–1 mg/L); PCT (>72 h) > 0.5–1 ng/mL (normal range 0.5–1 ng/mL).
    - Positive cultures of normally sterile body fluids.
  - Radiological signs of infection, including systemic infection, e.g.,
    - Gallbladder bed edema.
    - Unexplained pulmonary hypertension suddenly detected by echocardiography.

If any of the above-described temporary contraindications to FMT administration occur, the patient’s condition should be reassessed after a minimum of 6 h, and if the contraindications have resolved and do not recur within the next 6 h, the FMT procedure can be performed (in the study group) or a day identical to the day of microbiota administration (the so-called matching time point) can be set in the control group.

Assessment of the patient’s condition in terms of temporary contraindications to FMT administration and setting a date identical to the date of microbiota administration (the so-called matching time point):

- Physical examination.
- Follow-up laboratory tests:
  - If CRP 0.06–1 mg/L and/or PCT 0.6–1 ng/mL, follow-up in 6–12 h; follow-up imaging tests in approx. 6–12 h.
  - In the case of a central culture collection, wait at least 24–48 h for the result.

## 3. Materials and Equipment

Potential material donors for FMT are:

Healthy pregnant women from a group of at least 20, who consented to the collection of material from them, were positively screened around 20 weeks of pregnancy. The screening process included a detailed health questionnaire.

FMT material will be collected around 32 weeks of pregnancy from previously selected individuals and positively qualified in accordance with the donor qualification protocol, as recommended internationally and outlined in the proprietary method developed by Human Biome S.A. [29].

The conditions for the release of gut microbiota products are:

- A negative smear test for Streptococcus agalactiae (group B streptococcus, GBS) before the start of material donation (around the 20th week of pregnancy) and at 35–37 weeks of pregnancy (routine testing of pregnant women for GBS).
- Full-term delivery (full-term pregnancy), i.e., between 37 + 0 and 41 + 6 weeks of pregnancy.
- No perinatal complications—confirmed on the basis of a telephone interview with the donor.
- No reported health problems after delivery in the mother and child—confirmed on the basis of a telephone interview with the donor.

Due to the proven effectiveness of transplants from unrelated donors [30], the donor of choice in the experiment will be an unrelated donor who meets all the criteria necessary to ensure the biological safety of the material administered to recipients.

The material will be supplied in 8-millilitre syringes. No additional medical equipment is required.

## 4. Detailed Procedure

### 4.1. Transplant Recipients Procedures

FMT will be administered to patients in the study group who meet the inclusion criteria and do not meet the exclusion criteria, in the form of a deep rectal infusion, in a volume of 5 mL, twice at 6 h intervals, between 3 and 6 days after birth and/or up to 14 days in patients who experience temporary contraindications to the use of FMT or in patients who will be transferred to our center for treatment from an external facility.

In the first phase of the experiment, 10 patients from the study group born between 28 0/7 and 36 6/7 will undergo the procedure. All adverse effects of the therapy will be monitored and documented on an ongoing basis. If no serious adverse events (SAEs) occur, we will include another 10 patients born between 24 0/7 and 27 6/7 in the study (phase two of the experiment).

A serious adverse event (SAE) is defined as any medical event that, at any dose, meets at least one of the following criteria:

- Leads to death.
- Is life-threatening to the participant.
- Requires hospitalization or prolongs hospitalization.
- Leads to permanent or significant impairment of function or disability.
  A detailed description of the monitored parameters is provided below.

### 4.2. Protocol for Administering Fecal Microbiota Preparation

1. Check whether the patient is eligible for FMT therapy according to the inclusion criteria.
2. Assessing possible contraindications to FMT therapy (according to the exclusion criteria).
3. Discuss the therapy with the patient’s parents/guardians and obtain their consent for the child to participate in the experiment. Also obtain consent for the processing of personal data and the use of biological material samples for scientific purposes, as well as for participation in surveys and follow-up on the therapy’s effectiveness.
4. Collection of blood inflammation parameters (leukocyte count, CRP and procalcitonin) at the “point 0” stage, i.e., before the administration of microbiota.
5. Ensure that biological material samples are collected before (point “0”) FMT (feces, urine and blood samples), as described in the section below. Alternatively, collect samples before administering FMT.
6. Familiarize yourself with the characteristics of producing donor intestinal microbiota preparations in the form of a suspension (MBiotix^®^ HBI; Warsaw, Poland) and with the procedure for administering the therapy to patients.
7. Verify the presence of temporary contraindications to FMT administration.
8. Obtain the FMT preparations from the storage location.
9. Thaw the suspension in accordance with the characteristics of the FMT suspension production service and/or the manufacturer’s instructions. The suspension does not require special thawing conditions; allow the syringe to thaw in an uncovered container at an ambient temperature of 22–25 °C for approximately 2–2.5 h.
10. Administer two doses of intestinal microbiota (FMT1 and FMT2).

Due to the high safety profile of this form of FMT administration, patients will receive the preparation via deep rectal infusion using a soft Foley catheter.

The catheter will be connected to a syringe containing the suspension, and its lumen will be filled with the suspension to minimize the risk of oxygen entering the intestinal lumen.

The Foley catheter will then be inserted into the rectum to a depth of approximately 10–15 cm. After placement, 5 mL of the preparation (from a syringe containing 8 mL of the suspension) will be slowly administered directly into the catheter. Ultrasound guidance will be used to confirm the correct position of the Foley catheter and ensure the preparation is administered to the correct location—the cecum area.

After 5 mL of suspension has been administered, up to 2 mL of saline solution should be administered into the catheter for rinsing (depending on the size of the catheter). A certain volume of the product (approximately 3 mL) will remain in the syringe containing the suspension.

This procedure will be performed twice, at 6 h intervals (FMT1 and FMT2).

After each dose (FMT1 and FMT2), the catheter should be kept in the rectum for up to five minutes, and the patient should be placed in the Trendelenburg position and turned from side to side to ensure the preparation is distributed evenly throughout the intestine.

### 4.3. Monitoring the Transplant Recipients After the Procedure

Based on experience with FMT, the highest risk of developing sepsis, a rare but dangerous complication, occurs within the first six hours after the preparation is administered. Therefore, after the procedure, the patient will be continuously monitored.

The following will be assessed for the next 72 h after FMT administration:

1. The number of episodes of oral feeding intolerance, diarrhea and the presence of blood, mucus or pus in the stool.
2. The appearance of symptoms of infection or significant changes in vital signs (ECG, heart function and saturation—continuous measurement; blood pressure—4 times a day; temperature).
3. The number of cases of NEC developed.
4. Observation of other symptoms, such as convulsions, inconsolable crying and anxiety.
5. The number of positive blood cultures and the percentage of antibiotic therapy use.
6. Number of serious adverse events (SAEs), e.g., death, serious threat to health and/or life, and/or deterioration of health.

Blood inflammation parameters (leukocyte count, CRP and procalcitonin) will be collected before administration of the preparation (FMT1) and no later than 6–12 h after administration of FMT2, then every 12–24 h up to 72 h after FMT, depending on the patient’s condition.

A blood culture will be taken in the event of a significant increase in inflammatory parameters.

If any of the above-described criteria for temporary postponement of microbiota administration occur between FMT1 and FMT2 but resolve within 6 h (reassessment of the child’s condition after a minimum of 6 h) and do not recur within the next 6 h, the second dose of microbiota (FMT2) may be administered.

### 4.4. Procedures for the Patients in the Control Group

1. Check whether the patient meets the inclusion criteria for the experiment.
2. Check whether the patient meets the exclusion criteria.
3. Discuss the purpose and procedures of the experiment with the patient’s parents/guardians and obtain their informed consent for the child’s participation in the experiment. This includes consent for the processing of personal data and the use of biological material samples for scientific purposes, as well as consent for participation in surveys and follow-up on the effectiveness of therapy.
4. Collect blood parameters indicating inflammation (leukocytosis, CRP, procalcitonin) at the “0” point (“BEFORE intervention”), between signing the consent form and the fifth day of the child’s life.
5. Ensure that biological material samples (feces, urine and blood samples) are collected before (point “0”) FMT, as described in the section below, or collect samples before the sixth day of the child’s life.
6. For the control group, the matching time point for administering gut microbiota doses and counting “post” days is assumed to be the sixth day of the child’s life.
7. From day 6 (the matching time point), any adverse events or deviations from the norm will be recorded, such as deterioration in general condition, appearance of symptoms of infection or increase in inflammation indicators.
8. Blood inflammation parameters (leukocytosis, C-reactive protein (CRP), procalcitonin) will be collected during the “post-matching time point” period at least once within 72 h of day 6, or more frequently if the patient’s clinical condition requires it, as assessed by the attending physician.

The following will also be recorded in both study groups:

- The number of days spent in hospital;
- Percentage increase in body weight (birth weight vs. weight at discharge);
- The number of days of antibiotic therapy received during hospitalization (monitoring until discharge from the hospital conducting the experiment).

### 4.5. Protocol for Collecting Biological Samples from Both Groups for Safety and Exploratory Analyses

Biological material samples will be collected from patients in both groups (study and control) before FMT1 and after FMT2 at the following time points: 0, 7, 14, 30, 60 and 120 days after FMT2. These samples will be used for therapy safety analysis and exploratory analyses (e.g., microbiota analysis). Ideally, these samples will be blood (1 mL), urine and stool samples.

The collection point “0” (PRE) for stool samples should, if possible, be the first stool passed after birth (meconium) and after signing the informed consent form. In the case of premature babies transported from other hospitals, the PRE stool sample should be collected from the first stool passed after obtaining the consent of the guardians to participate in the experiment. Post-FMT samples (“POST”) are samples collected after the second dose of microbiota (FMT2) has been administered, sequentially after 7 days (POST7), 14 days (POST14), 30 days (POST30), 60 days (POST60) and 120 days (POST120).

For the control group, it is assumed that the day identical to the administration of the second dose of microbiota (matching time point) for counting “POST” days is the 6th day of the child’s life.

When collecting feces, at least three (3) fecal samples should be collected from each time point into three separate containers. The sample size should be similar to the size of a cherry, if possible.

Blood and urine should be collected at the following points: 0 (BEFORE), POST14, POST30 and POST120. An effort should be made to correlate these collections with routine collections of material for testing at the clinic in order to minimize the number of procedures and treatments for the newborn within the acceptable time windows (Table 1).

For blood samples, at each of the above-mentioned time points (BEFORE, POST14, POST30, POST120), whole blood (volume 0.5–1.0 mL) and serum (minimum volume 2 × 100 µL = 2 × 0.1 mL) should be collected, and, if a larger volume of serum is obtained, as much of the obtained volume as possible should be secured, divided into 150 µL portions.

For urine samples, at each time point mentioned, one sample (volume 2 mL) should be collected, which should be divided into microtubes of 1 mL each.

Each sample/sample container should be labeled with the patient’s number, date of collection and time point, and then frozen (optimally at −80 °C).

## 5. Expected Results

### 5.1. Primary Outcome

The primary outcome is safety, assessed through adverse event reporting and comparison with the control group.

Examination of the safety profile includes analysis of all adverse events, including any deviations from the norm: deterioration in general condition, appearance of symptoms of infection, and increase in inflammation indicators.

### 5.2. Secondary Outcomes

The following parameters will also be analyzed:

- Duration of hospitalization.
- Weight gain (percentage increase from birth to discharge).
- Length of antibiotic therapy.
- Incidence of other infectious or inflammatory complications.

The collected biological material will also enable the exploratory analysis of microbiota composition and its changes throughout the experiment.

### 5.3. Feasibility and Missing Data Management

Most biological samples will be collected during routine hospitalization, which in this population typically extends for several weeks. Therefore, the majority of scheduled collections will overlap with standard clinical monitoring.

Based on institutional experience, we anticipate an attrition rate of approximately 15%. For safety endpoints, no imputation of missing data will be performed. Analyses will be based on available data. The extent and reasons for missing data will be reported descriptively. Sensitivity analyses may be conducted if the proportion of missing data exceeds 10%.

### 5.4. Discussion

For many years, necrotizing enterocolitis has been associated with high and stable mortality rates, as well as severe complications. Despite significant progress in neonatal care, diagnosing and treating this condition remains a major clinical challenge. The available treatments are often ineffective and have side effects. Based on available studies, it is becoming increasingly clear that disturbances in gut microbiome composition play a very important role in the pathogenesis of NEC. The promising approach of restoring microbial balance using FMT could be beneficial in preterm infants at risk of NEC. Consistent experimental research shows reduced NEC incidence and reduced inflammation parameters following FMT. Although large-scale trials remain unavailable, early clinical studies suggest favorable safety, even among heavily immunosuppressed adult patients [31].

At the same time, meta-analyses do not provide clear evidence supporting the preventive effect of standardized probiotics against NEC. Although strains of Bifidobacterium and Lactobacillus may have beneficial effects, they appear insufficient to restore the natural microbiota, particularly in the context of multifactorial dysbiosis [12,13,14].

Despite numerous studies and meta-analyses evaluating probiotics in reducing NEC-related mortality and morbidity in preterm infants, ESPGHAN and AAP recommendations remain cautious. An optimal strain, dose, or combination for routine supplementation has not yet been clearly established [15,16,17,18].

We hope that this study will generate essential safety data to inform future randomized controlled trials assessing prophylactic efficacy and potential long-term benefits of FMT.

We acknowledge several important limitations of this study.

First, the open-label design may introduce detection bias, particularly for subjective safety endpoints such as feeding intolerance. Although objective definitions for sepsis and NEC (Bell’s criteria, CRP levels, blood cultures) are applied to mitigate this risk, complete elimination of assessment bias cannot be ensured.

Second, the non-randomized allocation of participants may introduce selection bias and residual confounding. Despite applying identical inclusion and exclusion criteria and conducting parallel observation in both groups, unmeasured baseline differences may influence observed safety outcomes. Therefore, causal inferences regarding between-group differences should be interpreted with caution.

Third, the relatively small sample size (20 participants per group) limits statistical power and increases the likelihood of type II error. The study is not powered to detect small or moderate differences in safety outcomes, and confidence intervals may be wide. Consequently, non-significant findings should not be interpreted as evidence of the absence of effect. The primary aim of this investigation is to generate preliminary safety data and identify potential safety signals to inform the design of future adequately powered randomized controlled trials.

Despite these limitations, the prospective design, standardized GMP-based FMT preparation, and structured safety monitoring strengthen the internal consistency of the study. We anticipate that the findings will provide essential groundwork for subsequent randomized efficacy trials.

## Figures and Tables

**Table 1:** Time windows for biological sample collection points.

Sampling Point	Sampling Time	Time Window ***
PRE (point “0”)	First stool after birth *	before administration of the first dose of microbiota (FMT1)
POST7	7 days after FMT2 ** 13th day of life (control group)	±3 days ***
POST14	14 days after FMT2 ** 20th day of life (control group)	±3 days ***
POST30	30 days after FMT2 ** 36th day of life (control group)	±7 days ***
POST60	60 days after FMT2 ** 66th day of life (control group)	±7 days ***
POST120	120 days after FMT2 ** 126th day of life (control group)	±14 days ***

* For preterm infants transported from other hospitals, the PRE stool sample should be obtained from the first stool passed after obtaining parental/guardian consent. ** For the intervention group, counting of “POST” days begins on day 1 after FMT1 administration (matching day). For the control group, counting begins from the infant’s chronological age: POST7 = 6th day + 7 days = 13th day of life, POST14 = 6th day + 14 days = 20th day of life, POST30 = 6th day + 30 days = 36th day of life, POST60 = 6th day + 60 days = 66th day of life, and POST120 = 6th day + 120 days = 126th day of life. *** Samples should be collected within the indicated time windows. If collection is not possible within the window, samples should be collected at the nearest possible time, and deviations must be documented.

## Data Availability

Data generated during this study will be publicly available upon publication of the results.
